# Facile *insitu* preparation of silver nanoparticles supported on petroleum asphaltene-derived porous carbon for efficient reduction of nitrophenols

**DOI:** 10.1016/j.heliyon.2022.e10659

**Published:** 2022-09-16

**Authors:** Hikmet Beyza Erdem, Sevil Çetinkaya

**Affiliations:** Kırıkkale University, Department of Chemistry, Yahşihan 71450, Kırıkkale, Turkey

**Keywords:** Porous carbon, Nitrophenol, Silver nanoparticle, Catalytic reduction, Pollutant

## Abstract

Herein, a facile *in situ* approach to synthesize catalytically active Ag nanoparticles supported on eco-friendly asphaltene-derived porous carbon (APC) was reported. Asphaltene-derived porous carbon was used as support for the first time for Ag@APC to prevent nanoparticles from aggregation, and then was evaluated as catalyst for the reduction of 4-nitrophenol (PNP), 2,4-dinitrophenol (DNP), and 2,4,6-trinitrophenol (TNP). The synthesized Ag nanoparticles were characterized by XRD, UV, BET, FESEM, TEM, and ICP analyses, revealing the formation of uniformly distributed, fcc structured crystalline Ag nanoparticles with BET surface area varied between 1500 and 1723 cm^−1^ with a porous carbon surface. Ag@APC nanocatalyst showed high catalytic efficiency in the reduction of nitrophenols in the presence of NaBH_4_ under mild conditions. The reduction of PNP, DNP, and TNP have pseudo-first-order rate constants of 0.3340, 0.2570, and 0.2408 min^−1^, respectively. The catalyst could be recyclable and reused for at least five successive runs without losing its original activity.

## Highlights

• Asphaltene-derived porous carbon (APC) was used as a support for Ag nanocatalyst (Ag@APC) for the first time.
• Facile in-situ preparation of Ag@APC catalyst.
• High catalytic efficiency (approximately 100%) in the nitrophenol reductions.
• High stability and reusability of Ag@APC catalyst.

## Graphical abstract

**Figure:**
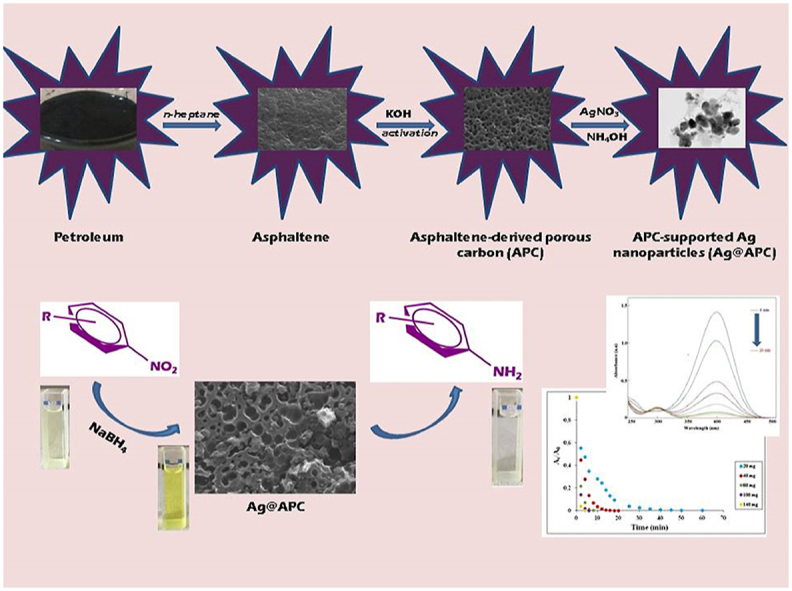

## 1 Introduction

Nitroaromatic compounds and their derivatives are widely used in various industrial and agricultural fields, including pharmaceuticals, papermaking, petrochemistry, fungicides, pesticides, preservatives, explosives, dyes, leather, and wood [1]. Nitrophenols commonly found in industrial wastewater have high toxicity, good solubility in water, high stability, and poor degradability [2]. Therefore, it is highly important to develop environmentally friendly, stable, and effective methods for removing these pollutants from industrial wastewater before its release into the environment [3]. An important method of removing nitrophenols (NPs) from industrial wastewater is their catalytic reduction to aminophenols (APs), which are used as intermediates in drug and dye synthesis [4]. The reduction of NPs to APs occurs with a metal catalyst accompanied by a reducing agent. The reduction of NP using a reducing agent without a metal catalyst is thermodynamically difficult. This indicates that reduction of the nitro group is not possible in the absence of metal catalyst [5]. Metal nanoparticles and their oxides have recently been examined as catalysts for the reduction of NP to AP [6]. Ag nanoparticles are widely used in catalytic applications due to their easy electron transfer [7]. The catalytic reduction of nitrophenols on the surface of Ag nanoparticles in the presence of sodium borohydride (NaBH_4_) is often expressed using the Langmuir-Hinshelwood adsorption model [8].

Silver nanoparticles are preferred to other metal nanoparticles for catalytic reactions due to their low-cost and high chemical activity [9]. Ag nanoparticles have distinctive physicochemical properties, including high electrical and thermal conductivity, surface-enhanced Raman scattering, catalytic activity, and nonlinear optical behavior [10]. Small-sized Ag nanoparticles generally show high catalytic activity for the reduction reactions because the higher surface-to-volume ratio and more negative redox potential are beneficial in electron transfer from the Ag surface to the reactants [11]. However, they tend to come together due to their high surface energies and van der Waals forces, which reduces the catalytic performance of Ag nanoparticles. Therefore, a certain amount of surface modifiers can be used during the synthesis process. In this case, organic modifiers can coat the surface and interact with Ag nanoparticles, leading to a marked reduction in their catalytic activity. To avoid these problems, Ag nanoparticles are loaded onto organic-inorganic supports. Graphene oxide, activated carbon, carbon nanofiber, N doped carbon, silica, metal oxide, zeolite, clay can be used to prevent aggregation. This approach result in the better catalytic performance of nanoparticles [12]. Supported Ag nanoparticles are not only stable in solution, but are easily separated and collected after the catalytic reaction [13]. Carbon supports have been attracting increased attention due to their cheap availability, stability in acidic-basic environments, and adjustable specific surface area and surface chemistry. Oxygen-containing functional groups on the activated carbon surface can act as anchoring sites during catalyst synthesis, which reduces hydrophobicity and thus improves catalytic performance [14]. Activated carbon-supported Ag nanoparticles prepared from various starting materials such as agricultural waste, bamboo, palm bark have been prepared and reported in the literature. The rate constant for conversion of NP to AP in these studies was in the range of 0.128–0.234 min^−1^ [14,15,16].

The catalytic reduction of NPs to APs in the presence of excessive NaBH_4_ is accepted as an environmentally friendly process due to the production of substances with low toxicity and high commercial value. Ag nanoparticles are economical catalysts that can effectively perform this catalytic reduction. The reduction performance of Ag nanoparticles can be enhanced by combining them with porous carbon, which prevents aggregation problems. Our aim in this study was to prepare high surface area Ag nanoparticles supported by porous carbon produced from asphaltene. Petroleum asphaltene was chosen as a raw material for porous carbon production due to its economic and environmental advantages (e.g. inexpensive, waste reuse, abundantly available, simplicity in obtaining asphaltene as raw material or waste). Here we also investigated the effectiveness of Ag nanoparticles for the catalytic reduction of 4-nitrophenol (PNP), 2,4-dinitrophenol (DNP) and 2,4,6-trinitrophenol (TNP). Various factors affecting the catalytic efficiency of the catalyst, including catalyst loading and initial contaminant concentration, have been studied in detail.

## 2 Experimental

### 2.1 Materials

Potassium hydroxide (KOH, ≥ 85%), silver nitrate (AgNO_3_, ≥99.5%), ammonium hydroxide (NH_4_OH, 30-33% NH_3_), hydrochloric acid (HCl(aq), 36.38%), 2,4-dinitrophenol (DNP, ≥98%) and n-heptane (≥99% HPLC) were provided by Sigma Aldrich, and 4-nitrophenol (PNP) and 2,4,6-trinitrophenol (TNP) were provided by Merck. Asphaltene was extracted from Turkish crude oil supplied from the West Raman region located in Batman. In the experiments, deionized water, produced by the Nuve ND 4 water distiller with storage tank, was used.

### 2.2 Characterization

X-ray diffractograms of the samples were obtained on a Rigaku Ultima-IV X-ray diffractometer (XRD), using Cu Kα radiation with a 2θ scan configuration in the range of 10–90°. A BET surface analyzer (Quantachrome Corporation, Autosorb-6) was used to measure nitrogen adsorption–desorption isotherm at 77 K. The BET surface area, total pore volume, and micropore area were obtained from the adsorption isotherms. The samples were sputter-coated with 5 nm gold-paladium and analyzed on a field-emission scanning electron microscope (FE-SEM; QUANTA 400F). The samples suspended in ethanol were left for stirring in ultrasonic treatment for 60 min. Then 1 drop of the mixture was deposited on a grid, dried for 1 night and transferred to a transmission electron microscopy (TEM). TEM images were obtained with a FEI TEM Tecnai G2 Spirit Biotwin high resolution transmission electron microscope (RTEM) with a lanthanum hexaboride (LaB_6_) electron gun, under accelerating voltage in the range of 20-120 kV. A PerkinElmer DRC II ICP-OES spectrometer was used for silver nanoparticle analysis. The concentration of nitrophenols remained after catalytic reactions was measured by using SHIMADZU UV-1800 UV–Vis spectrophotometer.

### 2.3 Synthesis of asphaltene-derived porous carbon (APC) by chemical activation method

APC was synthesized via a single-step pyrolitic process (two-step temperature programme). 4 g of KOH was dissolved in 10 mL of deionized water, and 1 g of asphaltene was added to it. The solution was mixed at a temperature of 60 °C for 2 h with a magnetic stirrer and then dried in a vacuum oven at 110 °C for 24 h to remove water. The dried sample was put into an alumina crucible under N_2_ gas at a flow rate of 84 mL/min, first heated to 450 °C with a heating rate of 10 ˚C/min, and waited for 2 h at this temperature. Secondly, the temperature was raised to 850 °C, and the sample was heated for 2 h. The sample was washed with 0.5 M of HCl solution and then deionized water up to the pH = 7 [17,18]. Finally, the sample was dried under vacuum at 105 °C for 24 h. The sample was donated as APC.

### 2.4 Synthesis of APC loaded Ag nanoparticles

Different concentrations of AgNO_3_ solutions were prepared for the synthesis of the APC-loaded Ag nanoparticles. 0.5 g of APC was added into an amber erlenmayer, then 10 mL of aqueous AgNO_3_ solution and 1 mL of concentrated NH_4_OH solution were added into the erlenmayer, respectively. The obtained mixture was mixed for 24 h with a magnetic stirrer. All of the samples were filtered, washed with distilled water, and dried to dryness [19]. The nanoparticles prepared with 0.01, 0.05 and 0.10 mol/L AgNO_3_ solutions were named as Ag(1)@APC, Ag(2)@APC and Ag(3)@APC, respectively.

### 2.5 Catalytic performance tests

The effect of the synthesized nanoparticles on the reduction of PNP, DNP and TNP in the presence of NaBH_4_ was examined. Although all synthesized catalysts were effective in reducing nitrophenols, Ag(2)@APC was chosen as a model catalyst for further catalytic tests due to its higher surface area. The initial nitrophenol concentrations and catalyst loadings on the reactions were investigated.

#### 2.5.1 Effect of initial nitrophenol concentration

A freshly prepared 10 mL (0.5 M) of NaBH_4_ solution and 40 mg of the catalyst were added into a 100 mL (0.10 mmol/L) of PNP solution and stirred at 400 rpm. 3 mL of the solution was taken and filtered for UV-Vis analysis. The reactions were repeated by changing the initial concentrations of PNP solution as 0.2, 0.3, 0.4 ve 0.5 mmol/L. For DNP and TNP reductions, 10 mL of 1.3 M NaBH_4_ and 1.64 M NaBH_4_ solutions were used, respectively.

#### 2.5.2 Effect of catalyst dosage

To a 100 mL (0.10 mmol/L) of PNP solution, 10 mL of 0.5 M NaBH_4_ and 20 mg of the catalyst were added, respectively. 3 mL of the solution was taken and filtered for UV-Vis analysis. The catalytic reactions were repeated by changing the catalyst loading as 40, 80, 100 ve 140 mg. For DNP and TNP reductions, 10 mL of 1.3 M and 1.64 M NaBH_4_ solutions were used, respectively. The percentage removal of NPs was calculated by the following Eq. (1).

(1) *E*(%) = [(*A*_*o*_−*A*_*t*_)/*A*_*o*_] x 100%

where E is the percentage removal efficiency, *A*_*o*_ is the initial nitrophenol absorbance, and *A*_*t*_ is the absorbance of nitrophenol at time t.

#### 2.5.3 Investigation of catalyst reusability

After each run of the catalytic reduction, the catalyst was separated and washed with water. Subsequently, the recovered catalyst was dried at 105 °C and re-used for the further catalytic reaction. The percentage of Ag in the recycled Ag(2)@APC was measured via the ICP method to evaluate the catalyst leaching.

## 3 Results and discussion

### 3.1 Synthesis of APC loaded Ag nanoparticles

**Figure 1:**
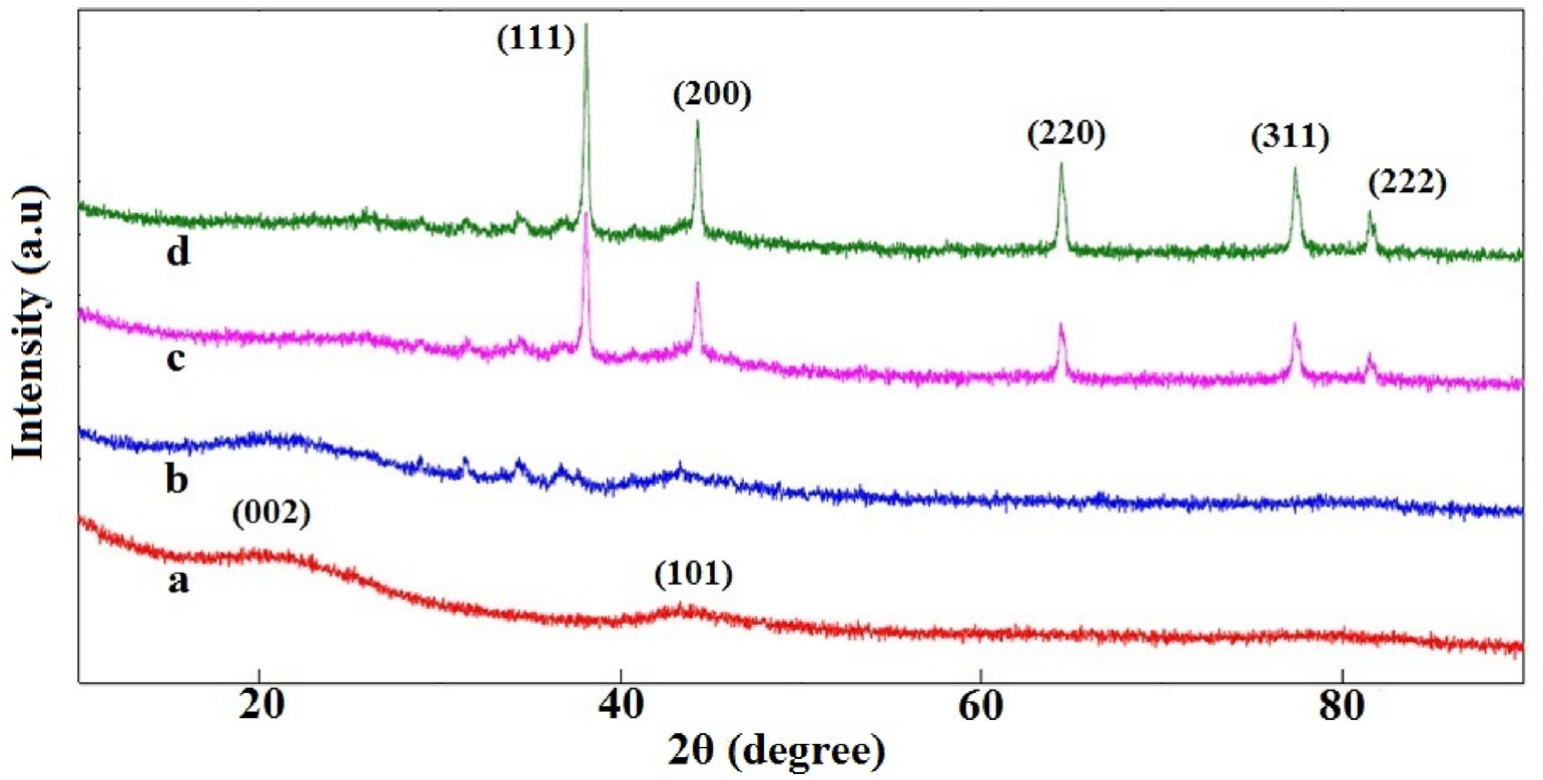
XRD patterns of (a) APC, (b) Ag(1)@APC, (c) Ag(2)@APC, (d) Ag(3)@APC.

In this study, Ag@APC nanoparticles were developed in the presence of NH_4_OH and it is thought that the formation of Ag nanoparticles was achieved by the chemical reactions reported previously [20]. The previously presented mechanism based on Tollen's synthesis method confirmed that in the presence of ammonia, Ag^+^ ions are reduced to Ag nanoparticles by aldehydes or carboxylates present on the porous carbon surface. Activated carbon surfaces have some aldehyde-type groups and some incompletely oxidized functionalities reacting with [Ag(NH_3_)_2_]^+^ to reduce Ag^+^ to the elemental form of Ag [21]. X-ray diffraction patterns of APC, Ag(1)@APC, Ag(2)@APC and Ag(3)@APC are shown in Figure 1. The XRD pattern of APC exhibited two broad diffraction peaks at 2θ of 20.99° and 43.60°, corresponding to the (002) and (101) planes [22]. These broad peaks indicated that the carbon structure was amorphous [23]. The diffraction pattern of supported nanoparticles showed peaks at 2θ of 38.08°, 44.27°, 64.38°, 77.34° and 81.43°. These observed peaks were assigned to face-centered cubic (fcc) crystal structure of metallic Ag nanoparticles (ICDD card no 00-004-0783) corresponding to (111), (200), (220), (311) and (222) planes [24]. These observations confirm the reduction of Ag^+^ to the metallic form. The average crystallite sizes of the Ag nanoparticles were calculated by using the data of the most intense peaks (111) of the XRD patterns and the Debye-Scherrer equation and the calculated average crystallite sizes of Ag nanoparticles were found between 41 and 94 nm. The absence of peaks attributed to silver oxides in the XRD patterns showed that the APC supported Ag nanoparticles were all in metallic form. The peak intensities related to Ag nanoparticles became proportionally higher by increasing the molar concentration of AgNO_3_ solution [25].

**Figure 2:**
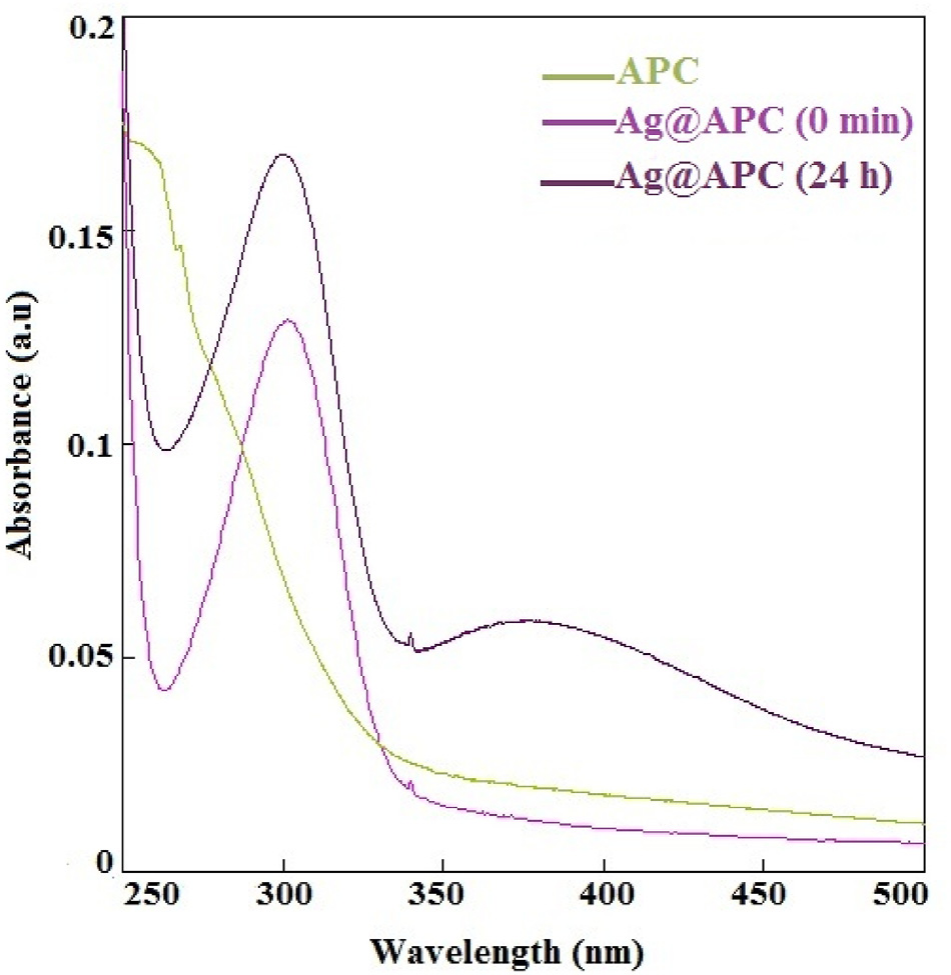
UV–vis spectra of Ag(1)@APC and APC.

Silver metal concentrations in Ag(1)@APC, Ag(2)@APC and Ag(3)@APC determined by ICP-OES were found to be 2.2 ± 0.1%, 6.0 ± 0.2% and 15.3 ± 0.1%, respectively. These results also confirmed that the amount of silver loaded on APC increased with increasing AgNO_3_ concentration. The existence of Ag in the Ag@APC nanoparticles was verified using a UV-Vis spectrophotometer and the obtained UV-Vis spectrum is presented in Figure 2. A broad absorption band was observed at 378 nm attributed to characteristic surface plasmon resonance (SPR) of Ag nanoparticles. They are typically known to exhibit a UV–Visible absorption at about 400 nm due to a collective oscillation of conduction electrons on the metal surface excited by light at specific wavelengths [26]. Observation of the broad band with higher wavelengths was due to the different sizes and shapes of the Ag nanoparticles and quantum size effect [27, 28].

**Figure 3:**
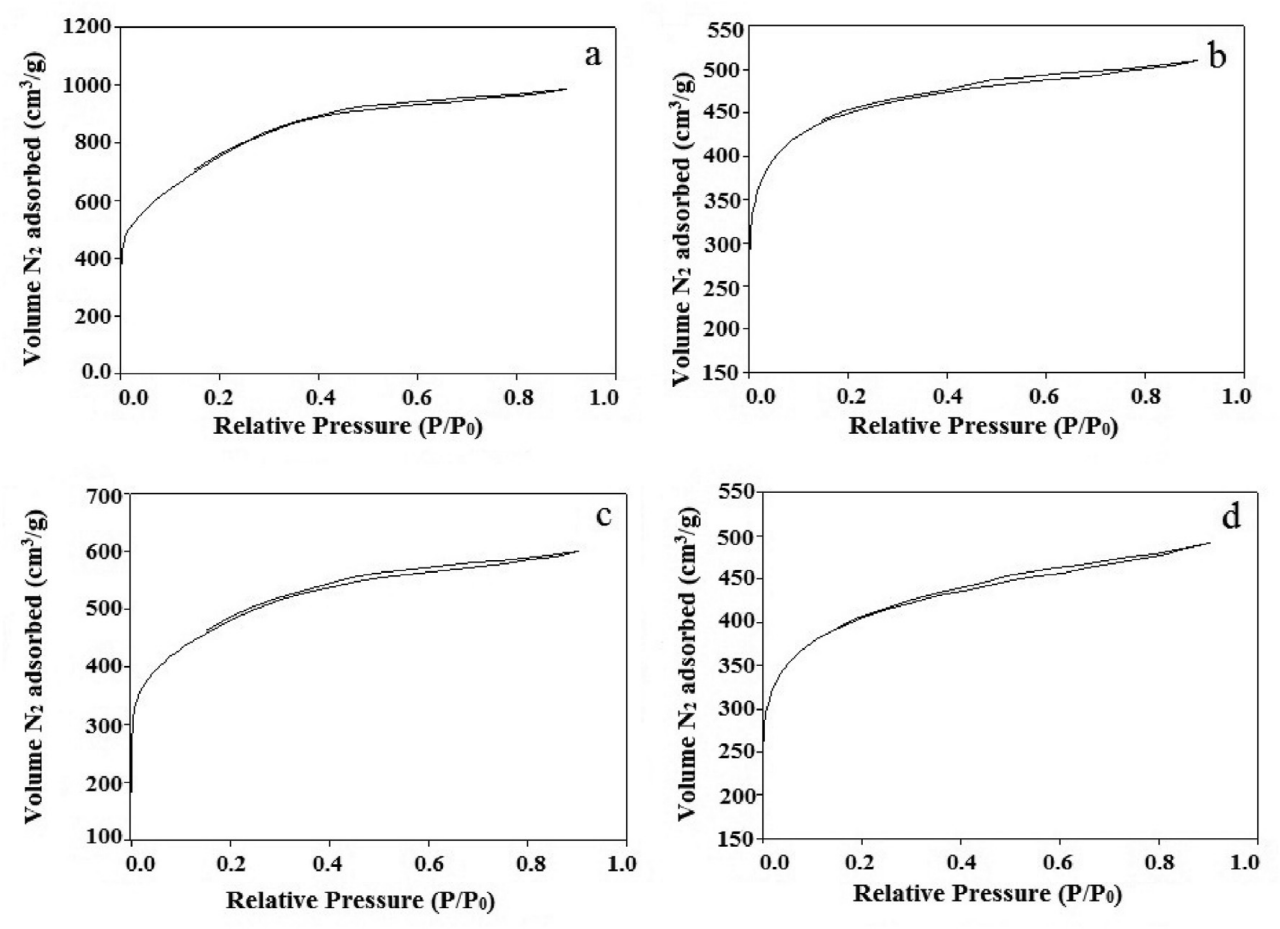
N_2_ adsorption-desorption isotherms of (a) APC, (b) Ag(1)@APC, (c) Ag(2)@APC, (d) Ag(3)@APC, at 77 K.

The detailed summary of the BET surface area, total pore volume, and pore diameter of all samples was presented in Table 1. The BET surface area obtained for Ag@APC nanoparticles was lower than that for APC. The 36-44% reduction of surface area was attributed to the direct blockage of pores by Ag nanoparticles, reducing the total surface area. However, BET surface area and total pore volume of Ag(2)@APC (1723 m^2^ g^−1^; 0.8937 cm^3^ g^−1^) were higher than those of Ag(1)@APC (1684 m^2^ g^−1^ and 0.7591 cm^3^ g^−1^) and Ag(3)@APC (1500 m^2^ g^−1^ and 0.7341 cm^3^ g^−1^). The APC and Ag@APC nanoparticles show similar adsorption-desorption isotherm as given in Figure 3(a-d). Based on the IUPAC classification, the isotherms are considered as type I. In these isotherms, N_2_ adsorption takes place rapidly at low relative pressures less than 0.1 P/P_0_. This observation confirms APC supported Ag nanoparticles are in microporous structure. The N_2_ adsorption takes place slowly at 0.1–0.9 P/P_0_ pressure values. It is the expected isotherm type for solids with microporous structure.

**Table 1:** Main chemical characteristics and textural properties of the synthesized APC and Ag@APC nanoparticles.

Sample	Physical parameters				
	S_BET_ (m^2^/g)	S_micro_ (m^2^/g)	V_pore_ (cm^3^/g)	V_micro_ (cm^3^/g)	Pore size (A)
APC	2276	873.7	1.3599	0.3728	6.225
Ag(1)@APC	1684	1522	0.7591	0.6392	6.125
Ag(2)@APC	1723	1429	0.8937	0.6634	6.225
Ag(3)@APC	1500	1269	0.7341	0.5411	6.125

S_BET_ was obtained by the Brunauer−Emmett−Teller method. S_micro_ and V_micro_ were calculated according to a t-plot analysis. V_pore_ was obtained by DFT method.

**Figure 4:**
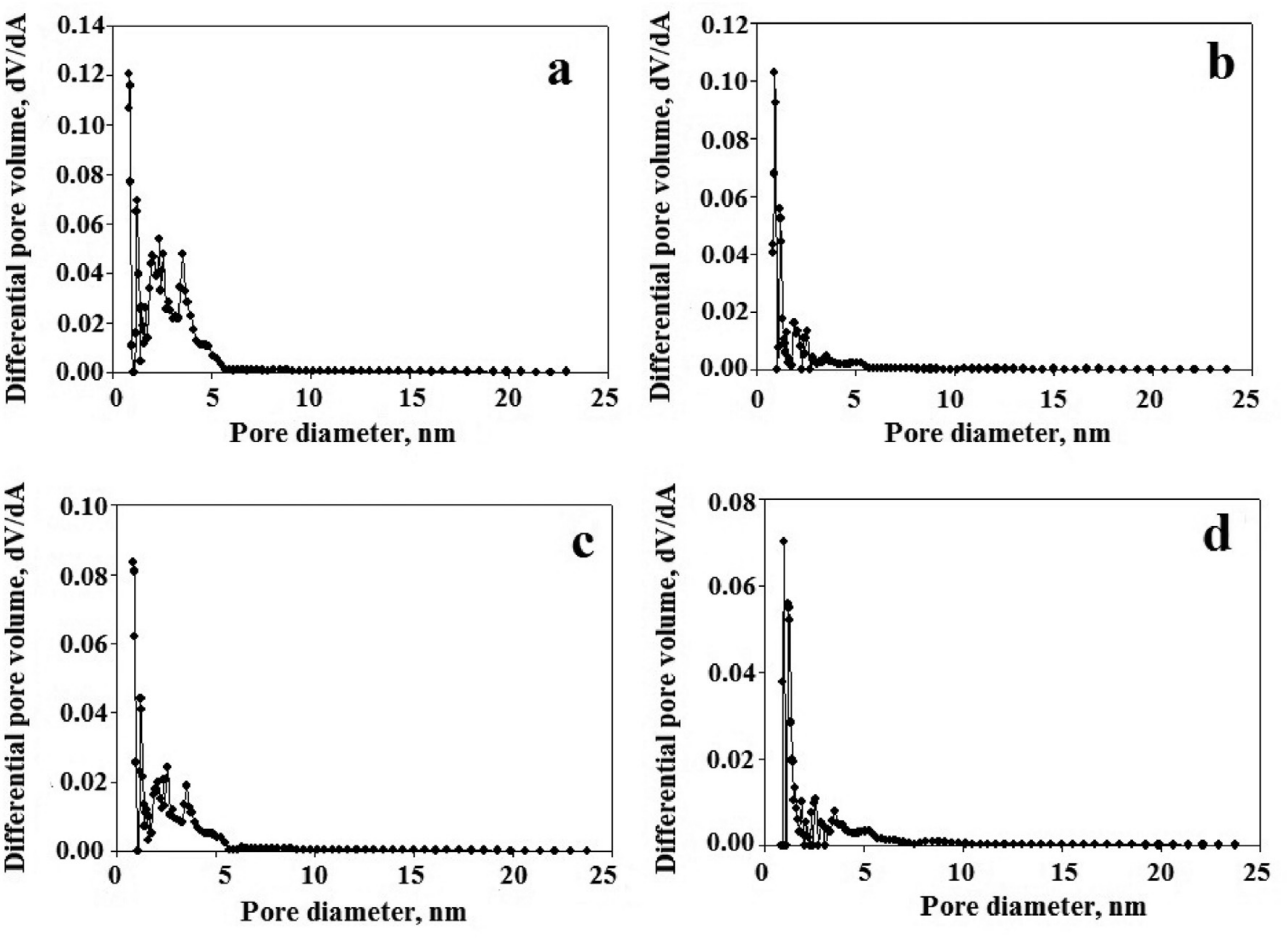
DFT pore size distributions of (a) APC, (b) Ag(1)@APC, (c) Ag(2)@APC, (d) Ag(3)@APC.

The pore size distribution graphs calculated by the DFT method of the synthesized samples are given in Figure 4(a-d). The graphs contain large-sized micropores (<2 nm) and small-sized mesopores between 1-5 nm. The microporous surface areas of APC, Ag(1)@APC, Ag(2)@APC and Ag(3)@APC samples are 1529, 1522, 1429 ve 1269 m^2^/g, respectively. The micropore surface area decreased with increasing silver concentration. The pore volume of Ag@APC nanoparticles also decreased when compared that of APC. While the pore volume of APC was 1.47 cm^3^/g, the pore volume of Ag@APC nanoparticles decreased to 0.74 cm^3^/g. The average pore diameters estimated for Ag@APC nanoparticles were between 6.125 and 6.225 A°.

**Figure 5:**
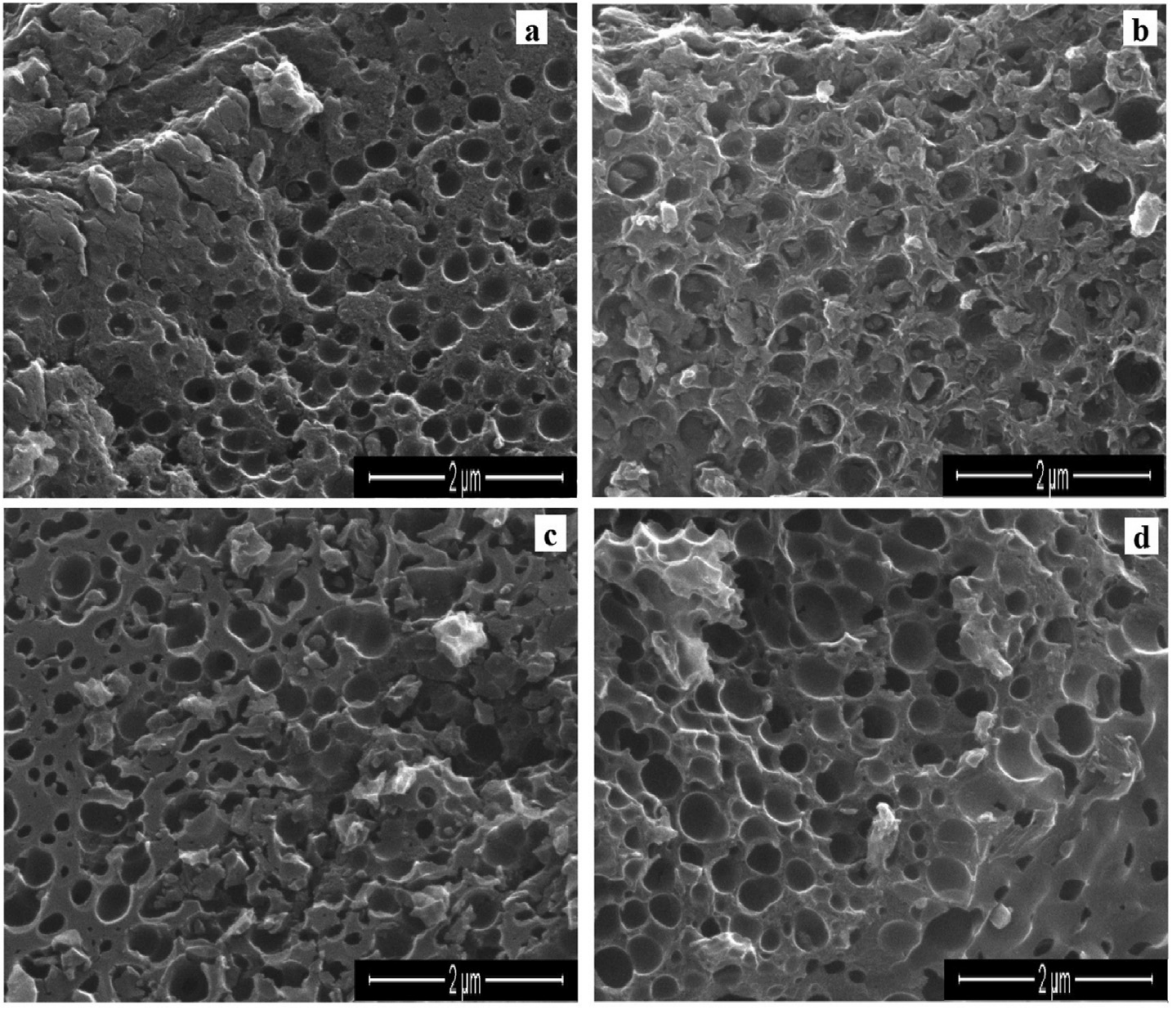
SEM images of (a) APC, (b) Ag(1)@APC, (c) Ag(2)@APC, (d) Ag(3)@APC at magnification of 50.000x.

**Figure 6:**
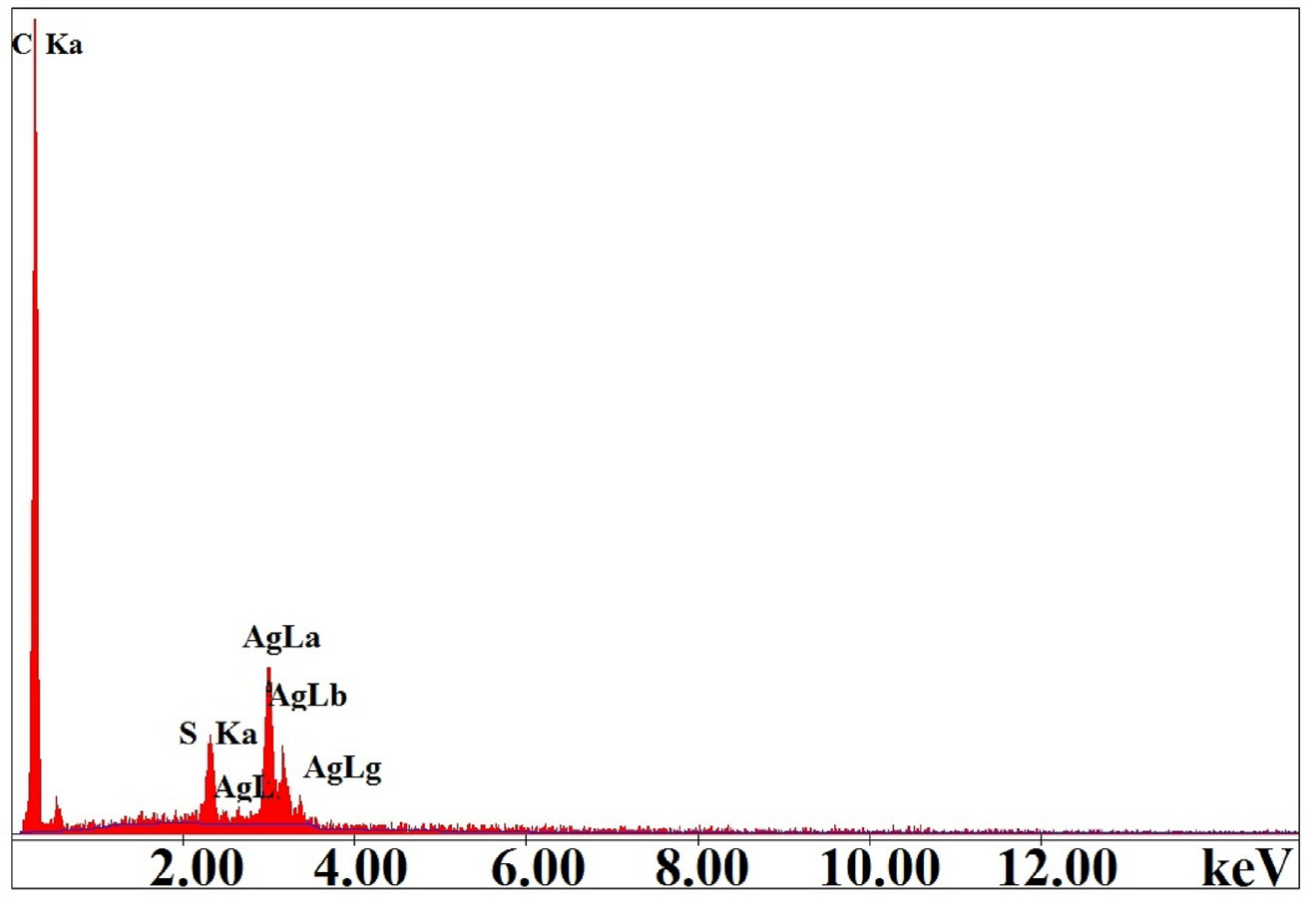
EDX spectrum of the synthesized Ag(2)@APC catalyst.

The distribution of an inorganic phase on the surface and pores of APC is seen in the SEM micrographs (Figure 5(a-d)). In these micrographs, the dominant phase forming the porous structures is APC. The existence of silver and carbon on the surfaces of Ag@APC nanoparticles was also varified by EDAX analysis and the EDAX spectrum for Ag(2)@APC is given in Figure 6. The strong optical absorption bands at 0.4 keV and 3 keV indicate the presence of elemental C and Ag in the Ag@APC nanoparticles due to the surface plasmon resonance, respectively [29]. Ag(2)@APC nanoparticle with 6 ± 0.2% Ag content was chosen to be used as a model catalyst in catalytic studies.

**Figure 7:**
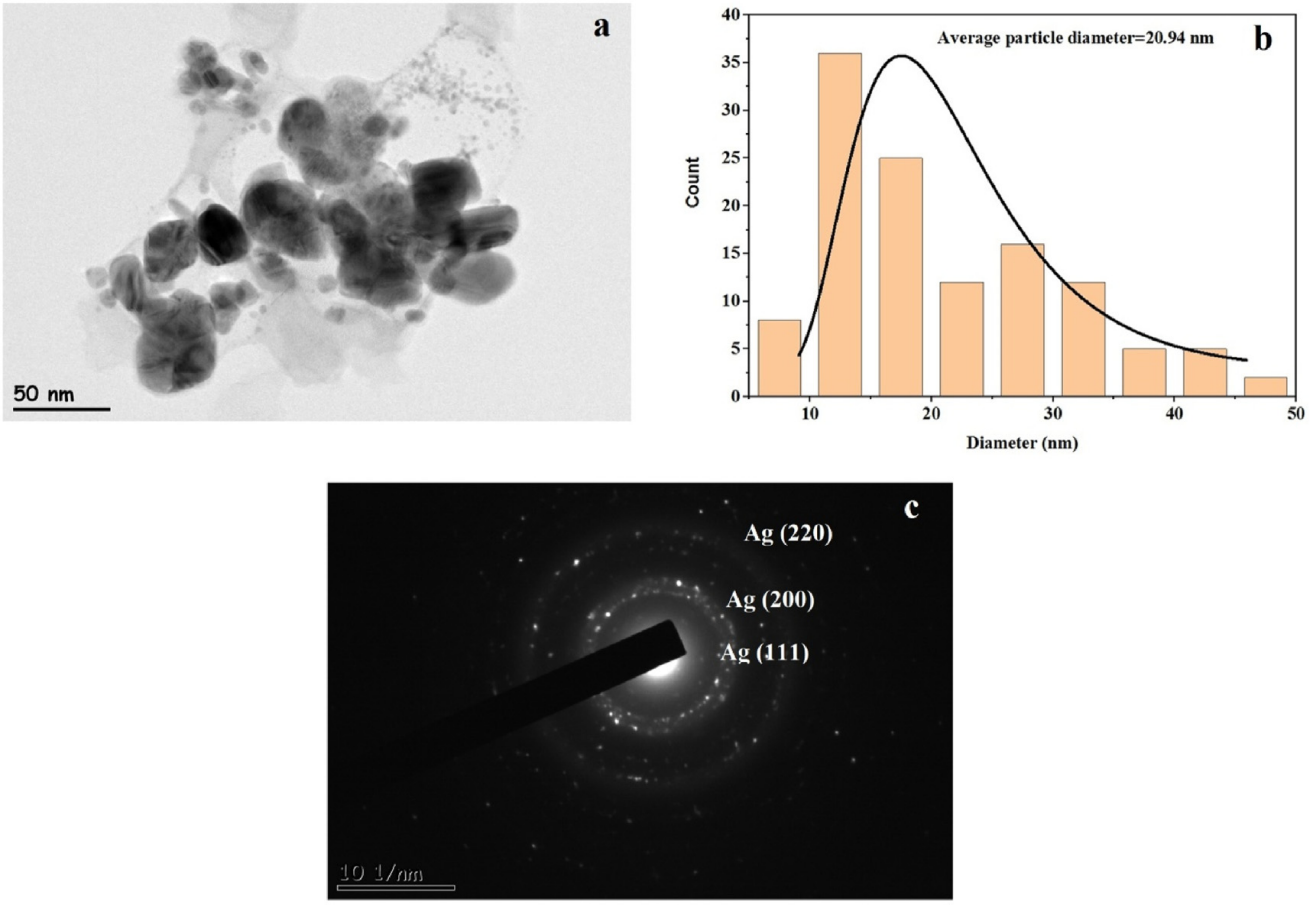
(a) RTEM image, (b) particle size distribution histogram, (c) SAED pattern of the synthesized Ag(2)@APC catalyst.

The surface morphology of the Ag(2)@APC used in the catalytic studies was examined by RTEM, and the RTEM photograph is given in Figure 7. The presence of Ag nanoparticle in the sample was evidenced by the dark black chunks appearing on the TEM image. The RTEM image (Figure 7(a)) and the particle size histogram (Figure 7(b)) of Ag nanoparticle supported on porous carbon show that the particle size ranges from 7 to 45 nm and possesses an average particle size of 21 nm. The presence of Ag nanoparticles can be seen from the selected field electron diffraction pattern (SAED) presented in Figure 7(c). This shows that the synthesized nanoparticle is in a crystalline structure [14].

### 3.2 Catalytic performance of Ag(2)@APC in the nitrophenol reduction by NaBH_4_

The catalytic activity of the prepared Ag(2)@APC nanoparticle was investigated by monitoring the reduction of nitrophenols (PNP, DNP, and TNP) in water.

#### 3.2.1 Catalytic reduction of PNP

**Figure 8:**
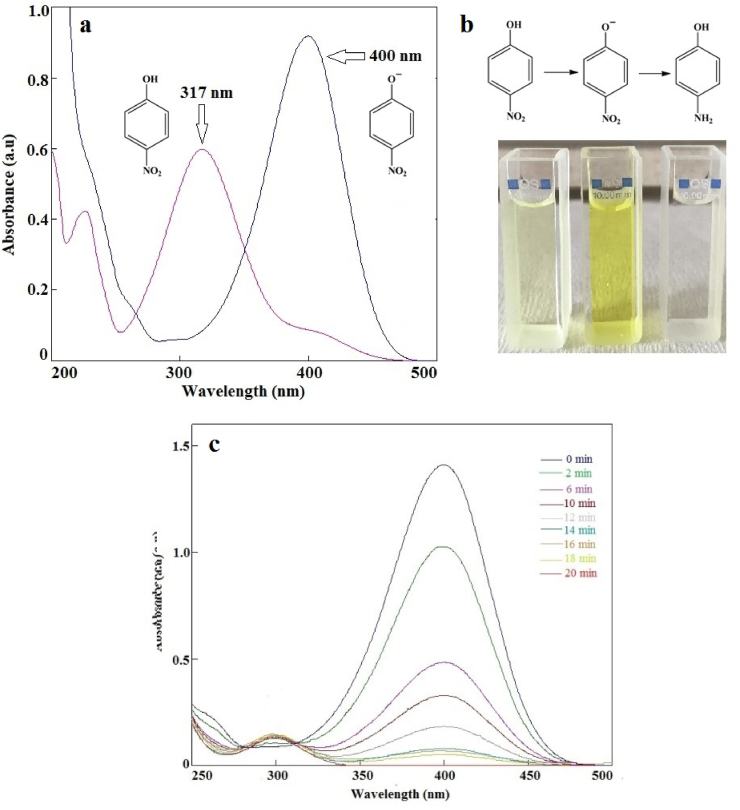
(a) UV-Vis spectra of PNP and 4-nitrophenolate ion, b) color change during the catalytic reduction of PNP to PAP, c) reduction of PNP to PAP in presence of Ag(2)@APC at time intervals. [PNP] = 0.1 mmol/L, [NaBH_4_] = 0.5 M, mcat = 40 mg, solvent = H_2_O, T = 25 °C.

The catalytic performance of the Ag(2)@APC was evaluated via the well-known reduction reaction of PNP in the presence of NaBH_4_ at room temperature. As shown in Figure 8(a-b), after the addition of NaBH_4_ to the reaction solution, the absorption peak (λ_max_) of PNP shifted from 317 nm to 400 nm, and the color of the solution changed from light yellow to deep yellow. This change was due to the increase in solution basicity and the formation of p-nitrophenolate ions in the reaction solution [14]. After adding the Ag(2)@APC catalyst, the intensity of the absorption peak at 400 nm started to decrease, while the characteristic peak of PAP appeared at 300 nm increased obviously Figure 8(c). The yellow color of the solution became colorless within 20 min. The isosbestic point at λ ∼ 300 nm indicates the complete reduction of PNP to PAP without side reactions [30]. The catalyst with catalytic activity accelerated the reduction process for PNP by stimulating the transition of electrons in NaBH_4_ to PNP [31].

**Figure 9:**
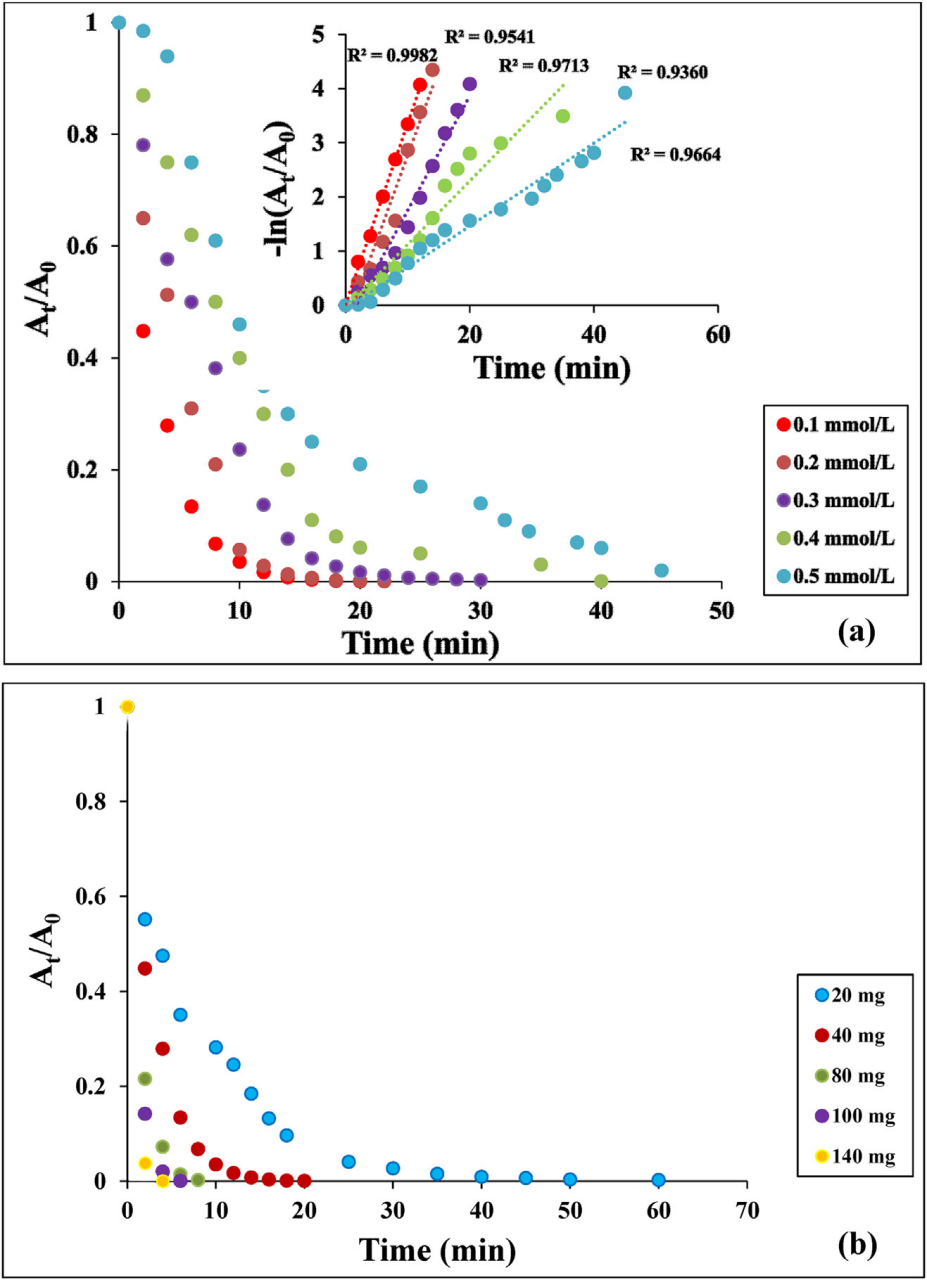
(a) Effect of initial phenol concentration on the reduction of PNP and plots of ln (C_t_/C_0_) versus reaction time. Reaction conditions: [NaBH_4_] = 0.5 M, m_cat_ = 40 mg, solvent = H_2_O, T = 25 °C, (b) Effect of catalyst loading on the reduction of PNP. Reaction conditions: [PNP] = 0.1 mmol/L, [NaBH_4_] = 0.5 M, solvent = H_2_O, T = 25 °C.

The effect of initial concentration of PNP was studied by varying the PNP from 0.1 to 0.5 mmol/L with a constant catalyst dosage of 40 mg and NaBH_4_ concentration of 0.5 M Figure 9(a) presents the time profiles of *A*_*t*_/*A*_*o*_ for the reduction of PNP using the Ag(2)@APC catalyst, where A_t_ and A_0_ are the absorbance values of 4-nitrophenolate ion at the reaction time t and 0, respectively. The conversion of PNP to PAP increased rapidly in the first 6 min and the time required for complete reduction was 20 min with a concentration of PNP of 0.1 mmol/L. The required time for the complete reduction increased from 20 to 45 min as the concentration of PNP increased from 0.1 to 0.5 mmol/L. The increase in time can be explained by the decrease in the electron transfer rate to the Ag(2)@APC surface, as the catalyst surface becomes fully saturated with the reactant molecules [32]. The reaction process followed the pseudo-first-order kinetics which can be described by the Eq. (2) as follows:

(2) ln *A*_*t*_/*A*_*0*_ = -k_1_t

Catalytic efficiency k_1_, the correlation coefficient (R^2^), and activity parameter were calculated from the plots of -ln(*A*_*t*_/*A*_*o*_) versus reaction time for each initial concentration of PNP, and are listed in Table 2. The corresponding rate constant decreased from 0.334 to 0.0766 min^−1^ as the initial concentration of PNP increased from 0.1 to 0.5 mmol/L. The effect of catalyst loadings was also investigated by recording the sequential reduction process of PNP by UV-vis spectroscopy at a designated time interval. Figure 9(b) shows the effect of catalyst loading (i.e., 20, 40, 80, 100 ve 140 mg) on the reduction in the presence of 100 mL of 0.1 mmol/L PNP solution and 10 mL of 0.5 mol/L NaBH_4_ aqueous solution. In these reductions, the fastest PNP reduction was achieved with the use of 140 mg catalyst. As the amount of catalyst increased, the required time for the complete conversion decreased. 99% removal efficiency was achieved in 4 min with 140 mg catalyst.

**Table 2:** Kinetic parameters for the reduction of phenols.

Phenol	Initial concentration (mmol/L)	Rate constant, k_1_ (min^−1^)	R^2^	Activity parameter, k/m (mg^−1^ min^−1^)	Efficiency (%)
PNP	0.1	0.3340	0.9982	0.0084	99.94
	0.2	0.3159	0.9541	0.0079	99.94
	0.3	0.2114	0.9713	0.0053	99.74
	0.4	0.1172	0.9360	0.0029	99.25
	0.5	0.0766	0.9664	0.0019	98.01
DNP	0.1	0.2784	0.9928	0.0070	99.83
	0.2	0.1448	0.9774	0.0036	99.02
	0.3	0.0879	0.9117	0.0022	99.07
	0.4	0.0678	0.9330	0.0017	98.54
	0.5	0.0646	0.9102	0.0016	98.86
TNP	0.1	0.2408	0.9963	0.0060	98.11
	0.2	0.1949	0.9921	0.0049	99.74
	0.3	0.0817	0.9341	0.0020	99.28
	0.4	0.0678	0.9387	0.0017	99.52
	0.5	0.0519	0.9463	0.0013	99.02

**Figure 10:**
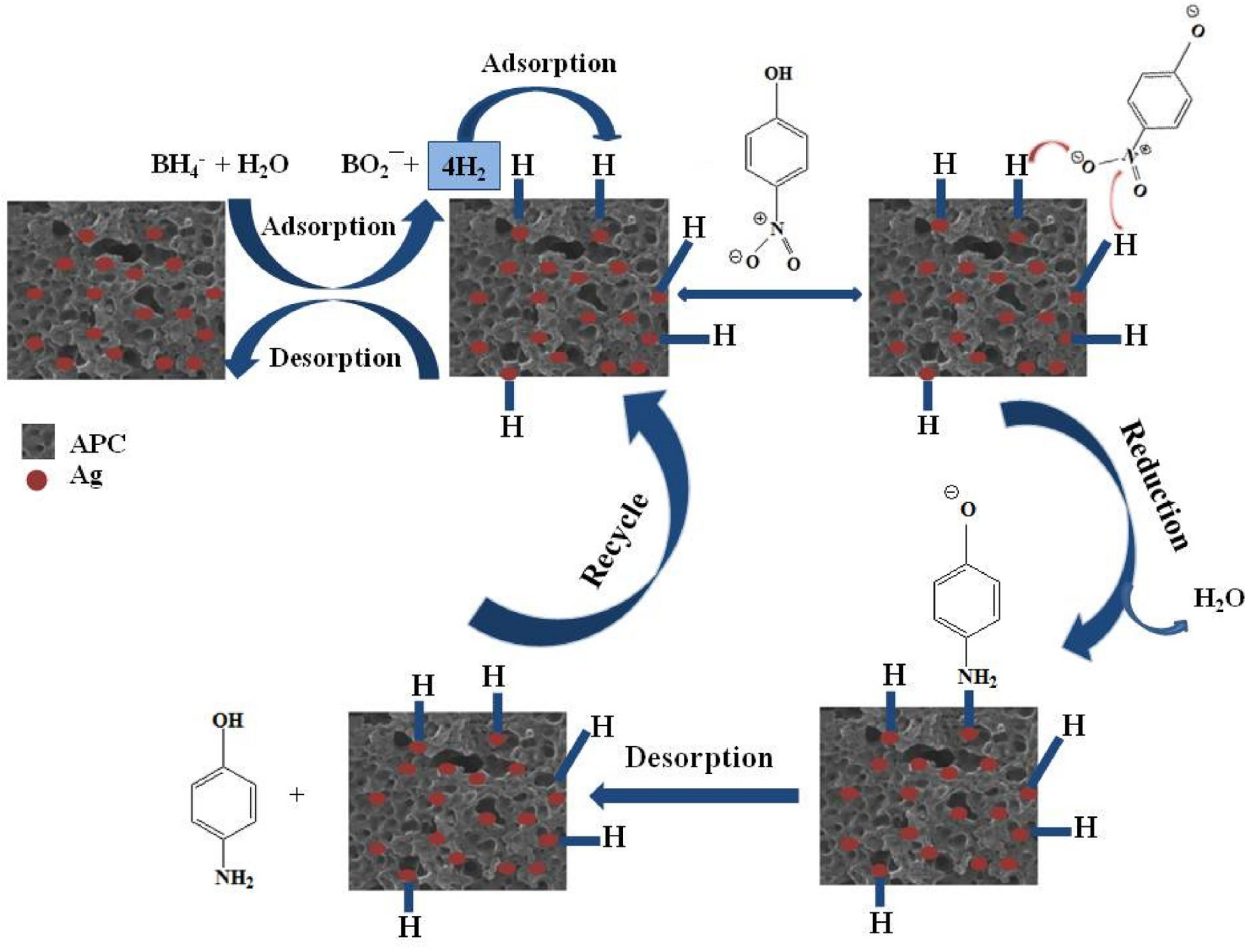
The reduction mechanism of PNP to PAP by NaBH_4_ using Ag(2)@APC catalyst.

A decrease in the rate constant with increasing concentration of PNP proves the reaction mechanism fits well Langmuir–Hinshelwood (LH) model [33]. The catalytic performance of the catalyst is mainly due to the repeated electron transfer between PNP and Ag nanoparticles during this reaction. The reduction mechanism of PNP to PAP by NaBH_4_ using Ag(2)@APC catalyst is proposed based on the LH model, as given in Figure 10. According to this model, BH_4_– ions are adsorbed on the surface of Ag(2)@APC catalyst. The hydrogen atoms are transferred to the surface of the Ag nanoparticles and the PNP molecules are adsorbed on the surface of the nanoparticles. Reduction of PNP occurs when the adsorbed PNP reacts with the hydrogen atoms attached to the surface of the nanoparticles and then is followed by the desorption of the product, PAP [34, 35].

#### 3.2.2 Catalytic reduction of DNP

**Figure 11:**
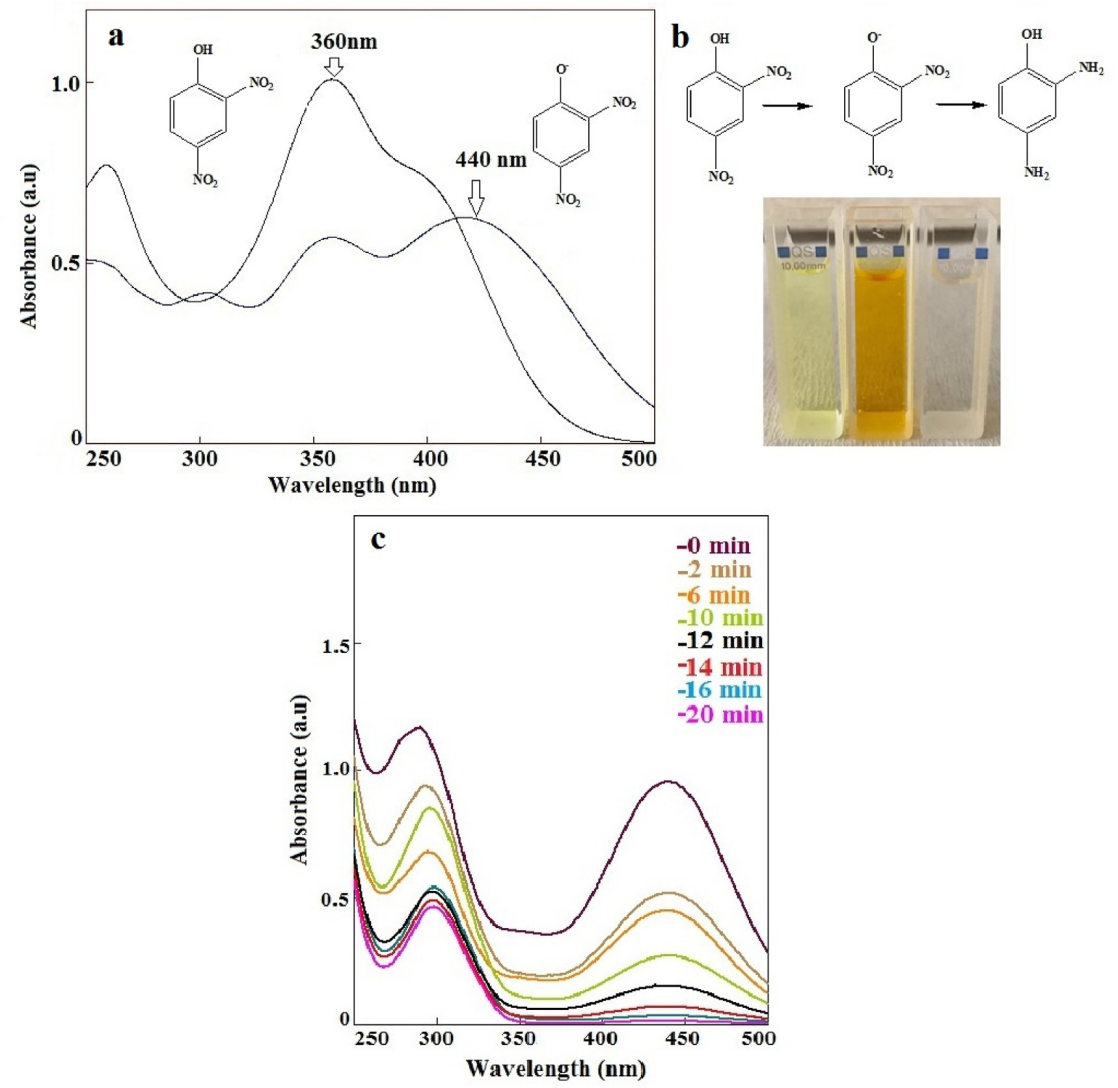
(a) UV-Vis spectra of DNP and 2,4-dinitrophenolate ion, (b) color change during the catalytic reduction of DNP to DAP, c) reduction of DNP to DAP in presence of Ag(2)@APC at time intervals. [PNP] = 0.1 mmol/L, [NaBH_4_] = 1.3 M, m_cat_ = 40 mg, solvent = H_2_O, T = 25 °C.

The catalytic activity of the synthesized Ag(2)@APC catalyst was evaluated by the reduction of DNP in water using NaBH_4_ as a reducing agent. UV–vis spectrum of DNP is seen in Figure 11(a), and has a maximum absorbance of 360 nm and a shoulder at a lower frequency of 396 nm. The addition of NaBH_4_ to the DNP solution resulted in shifting the absorption peak at 360 nm to 440 nm (redshift), and the yellow solution became orange (Figure 11(b)). The peak intensity at 300 nm related to the formation of 2,4-diaminophenol (DAP) started to increase by adding the catalyst to the reaction mixture, while the peak intensity of nitrophenolate ion at 440 nm was decreasing (Figure 11(c)).

**Figure 12:**
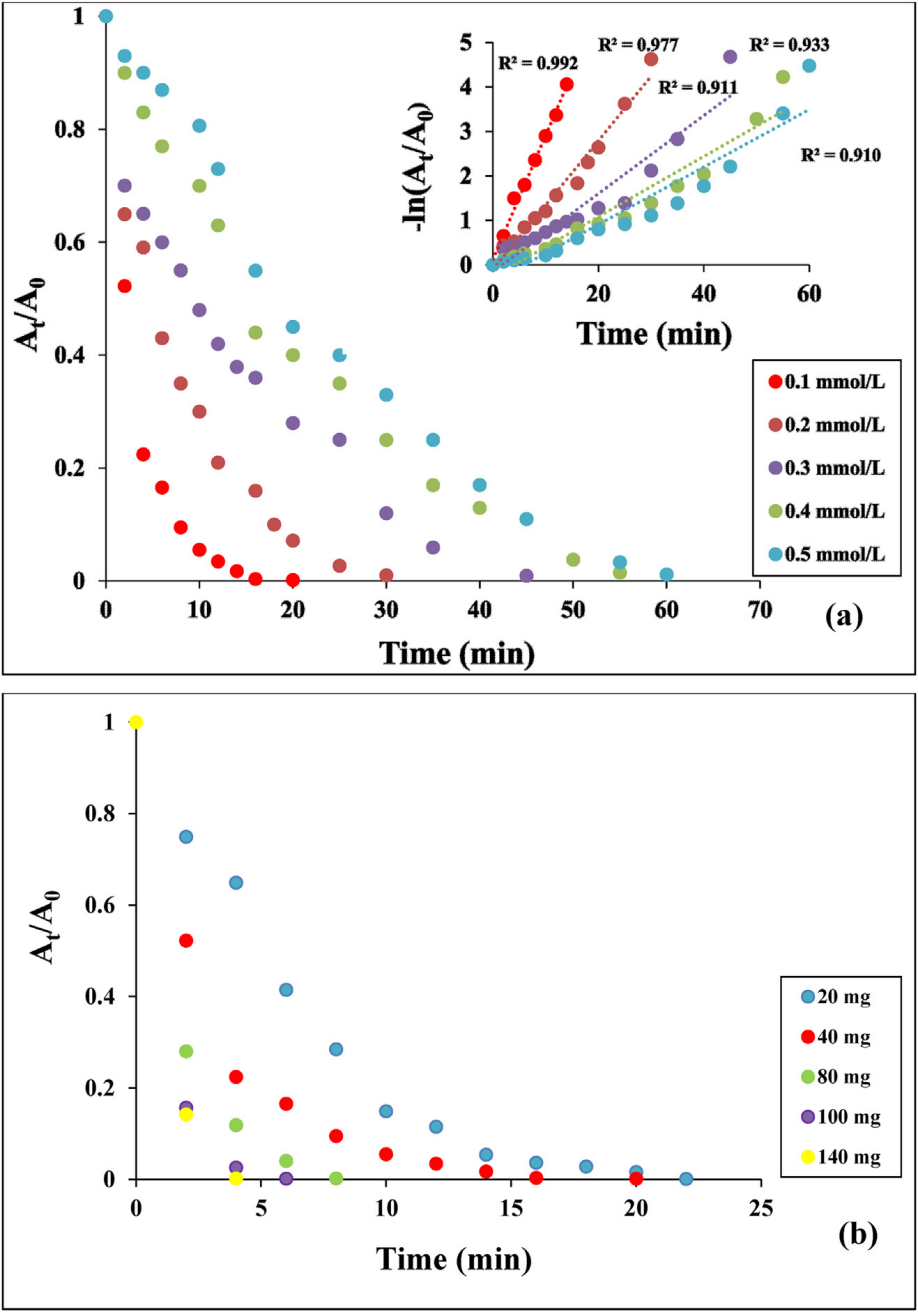
(a) Effect of initial phenol concentration on the reduction of DNP and plots of ln (C_t_/C_0_) versus reaction time. Reaction conditions: [NaBH_4_] = 1.3 M, m_cat_ = 40 mg, solvent = H_2_O, T = 25 °C, (b) Effect of catalyst loading on the reduction of DNP. Reaction conditions: [DNP] = 0.1 mmol/L, [NaBH_4_] = 1.3 M, solvent = H_2_O, T = 25 °C.

The solution color also became completely colorless. Reduction of DNP to DAP with NaBH_4_ is thermodynamically favorable as borohydride ions are the strong reducing agent in the aqueous medium. However, the reduction proceeds too slowly kinetically. The kinetic barrier arising from the potential difference between the donor (borohydride) and acceptor (nitrophenolate) ions leads to it [29]. The peak position and intensity did not changed without using the Ag(2)@APC catalyst. Both catalyst and NaBH_4_ are required to complete the catalytic reduction shortly. Nanoparticles catalyze the reaction by facilitating electron transfer from borohydride to 2,4-dinitrophenolate molecules [36]. The effect of initial DNP concentrations on the catalytic performance of Ag(2)@APC catalyst was examined in the range of 0.1–0.5 mmol/L. As given in Figure 12(a), the reduction efficiency decreased from 99 to 55% by increasing DNP concentration up to 0.5 mmol/L, in 20 min. As given in Figure 12(a), the plots of ln(*A*/*Ao*) vs. reaction time were linear, indicating that DNP reduction follows a pseudo-first-order reaction. The catalyst exhibited a reaction rate constant of 0.2784 min^−1^ and 0.0646 min^−1^ with an initial DNP concentration of 0.1 mmol/L and 0.5 mmol/L, respectively. The dosage effect on the catalytic reduction of DNP to DAP was examined by changing the catalyst dosage of 20, 40, 80, 100, and 140 mg of Ag(2)@APC nanoparticle in the presence of NaBH_4_ at 0-22 min, as shown in Figure 12(b). The total reduction time to DAP decreased as the amount of catalyst increased. The reduction of DNP was completed in 4 min when 140 mg of the catalyst was used. The increase in reaction rate was due to the increase in the number of active catalytic sites as the concentration of Ag nanoparticles increased.

#### 3.2.3 Catalytic reduction of TNP

**Figure 13:**
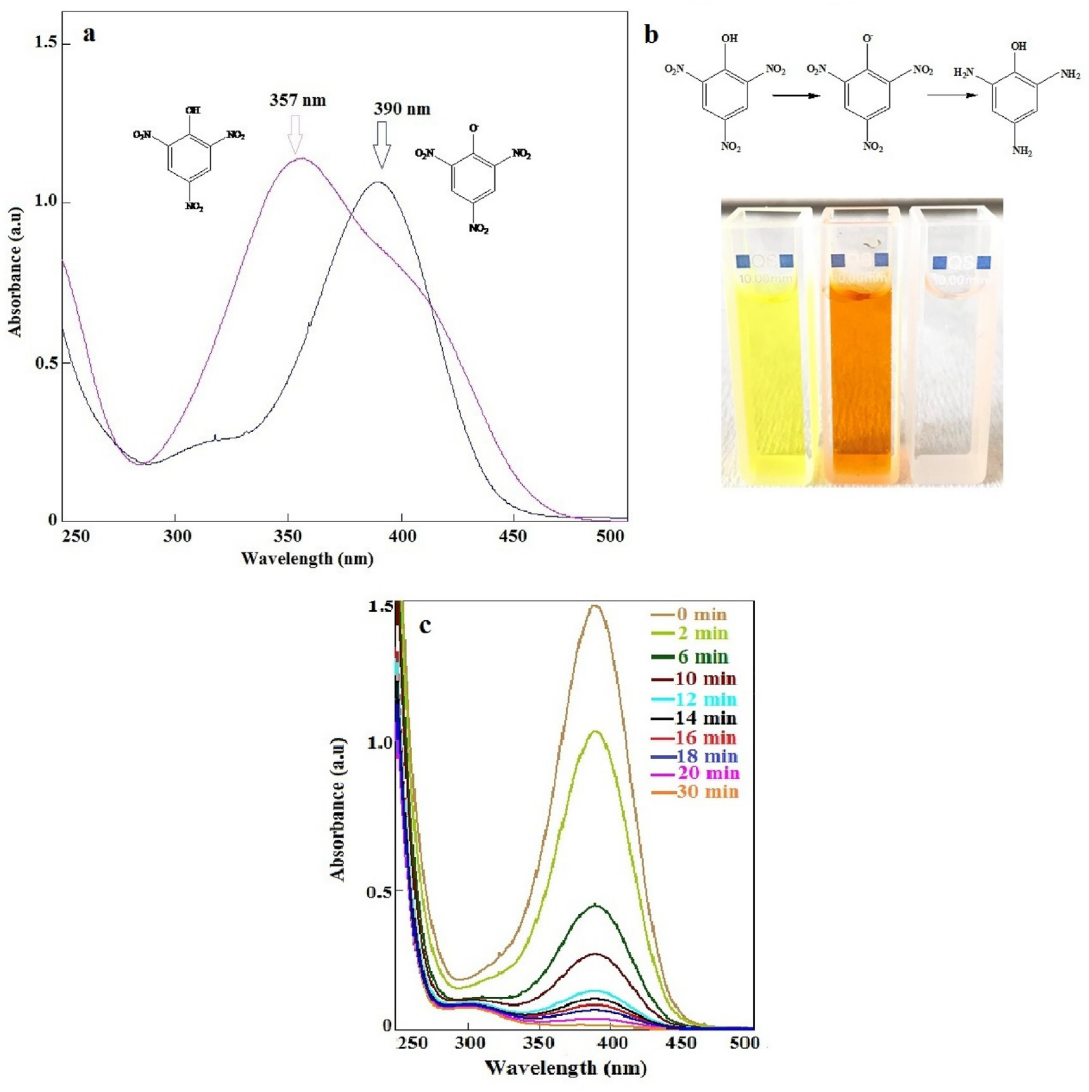
(a) UV-Vis spectra of TNP and 2,4,6-trinitrophenolate ion, (b) color change during the catalytic reduction of TNP to TAP, c) reduction of TNP to TAP in presence of Ag(2)@APC at time intervals. [PNP] = 0.1 mmol/L, [NaBH_4_] = 1.64 M, m_cat_ = 40 mg, solvent = H_2_O, T = 25 °C.

The catalytic performance of Ag(2)@APC was also investigated at the various initial TNP concentration and catalyst dosage. The maximum absorbance of TNP was detected at a wavelength of 357 nm as shown in Figure 13(a). The catalytic activity of Ag(2)@APC in reducing TNP was investigated in the presence of NaBH_4_. When the NaBH_4_ aqueous solution is added to the TNP solution, the absorption peak at 357 nm shifted to 390 nm (redshift), and the color of the solution changed from yellow to dark orange (Figure 13(b)). This was due to the increase in the basicity of the solution, and the formation of 2,4,6-trinitrophenolate ions [14].

In the catalytic studies, the absorbance measurements were taken at a wavelength of 390 nm to examine the reduction of TNP. After the catalyst was added to the reaction solution, the intensity of the absorption peak at 390 nm was greatly reduced, while a new absorption peak of 2,4,6-triaminophenol (TAP) was formed at 300 nm, and the solution became colorless (Figure 13(b-c)).

**Figure 14:**
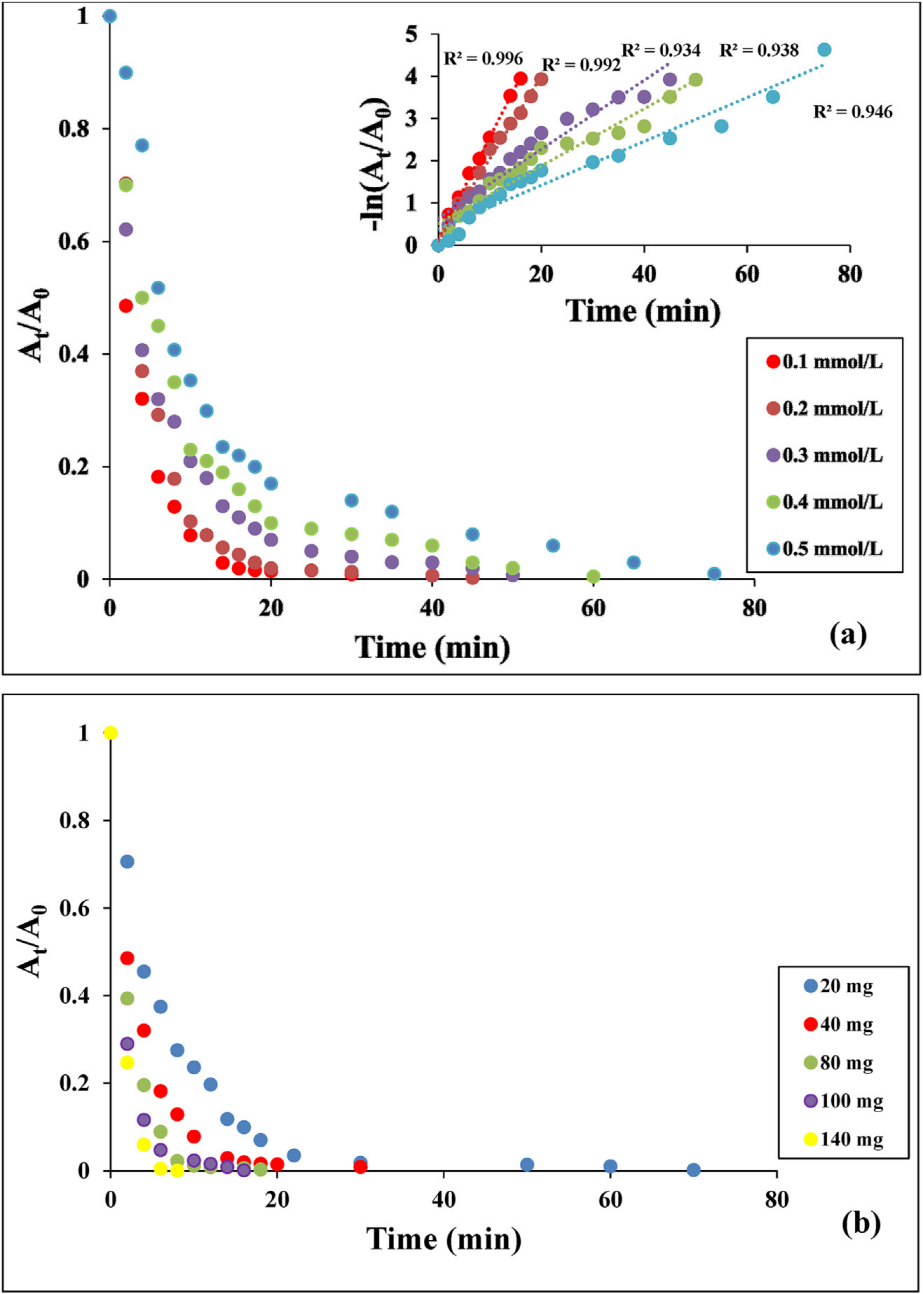
(a) Effect of initial phenol concentration on the reduction of TNP and plots of ln (C_t_/C_0_) versus reaction time. Reaction conditions: [NaBH_4_] = 1.3 M, m_cat_ = 40 mg, solvent = H_2_O, T = 25 °C, (b) Effect of catalyst loading on the reduction of TNP. Reaction conditions: [TNP] = 0.1 mmol/L, [NaBH_4_] = 1.64, solvent = H_2_O, T = 25 °C.

The catalytic performance of Ag(2)@APC was investigated at the various TNP concentrations (0.1–0.5 mmol/L) and catalyst loadings (20-140 mg). A_t_/A_0_-time and -ln(A_t_/A_0_)-time graphs are given in Figure 14(a-b). First-order rate constants for different initial concentrations of TNP were calculated from the slope of ln(A_t_/A_0_) versus time. The rate constants of Ag(2)@APC for the initial concentrations of TNP of 0.1 and 0.5 mmol/L were calculated to be 0.2405 min^−1^ and 0.0519 min^−1^, respectively. The results show that the reduction time to TAP increased from 30 min to 75 min when the TNP concentration increased from 0.1 mmol/L to 0.5 mmol/L. The results show that the reduction proceeds faster as the amount of catalyst loading increases, leading to a higher reaction rate as it provides more active sites for the reaction. The reduction of TNP was completed in 8 min when 140 mg of the catalyst was used. Ag(2)@APC nanoparticle shows excellent catalytic activity when compared to the other supported Ag catalyst materials reported in the literature [37].

#### 3.2.4 Recyclability of the catalyst

**Figure 15:**
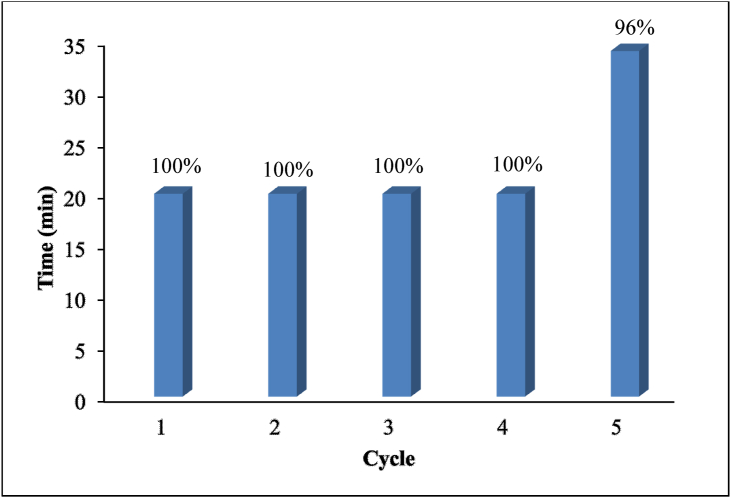
Catalyst reusability studies on the Ag(2)@APC catalyst. Efficiency is labeled as a percentage for each cycle. Reaction conditions: [PNP] = 0.1 mmol/L, m_cat_ = 40 mg, solvent = H_2_O, T = 25 °C.

**Figure 16:**
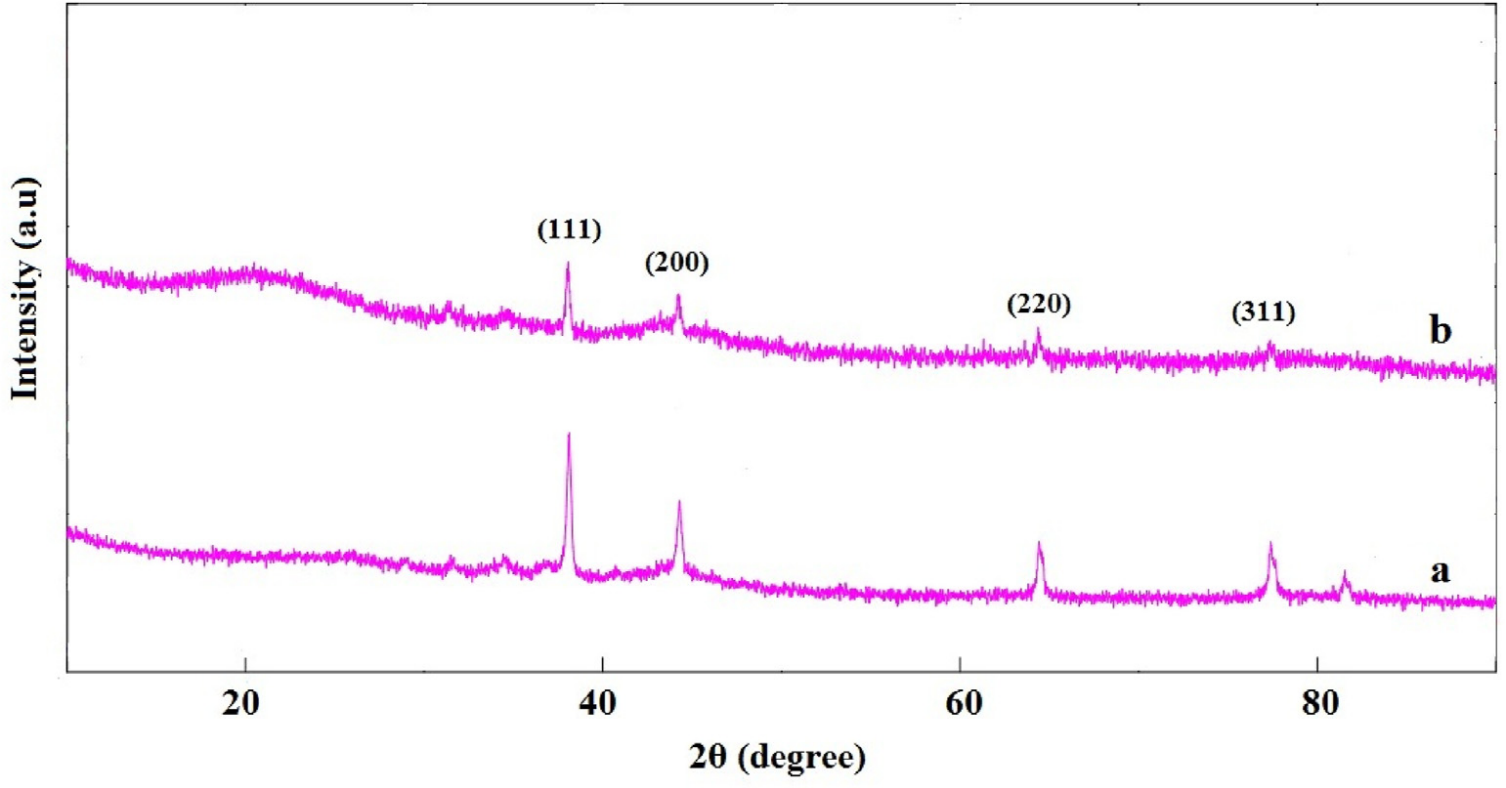
XRD patterns of (a) fresh Ag(2)@APC catalyst, (b) recycled Ag(2)@APC catalyst after 5^th^ cycle. Reaction conditions: [PNP] = 0.1 mmol/L, m_cat_ = 40 mg, solvent = H_2_O, T = 25 °C.

Since the recyclability is an important aspect for heterogeneous catalyst, five successive cycles of PNP reduction using Ag(2)@APC catalyst was further investigated to check the recyclability of the catalyst. The Ag(2)@APC catalyst was easily separated from the reaction mixture by filtration and reused under the same reaction conditions. After each run, the catalyst was filtered, washed for several times with distilled water and then dried under vacuum oven. Figure 15 shows catalyst efficiency vs. the time required for the complete reduction. The reduction was completed within 20 min up to the first 4 cycles, while the reduction was completed within 34 min in the 5^th^ cycle. Hence, the catalyst could be successfully reused up to the 5^th^ cycle without affecting the efficiency of the catalyst. No Ag leaching occurred even upon the 5^th^ run of the reaction. Acidic functional groups (i.e carboxyl, anhydrides, phenol, lactone, and lactol) are usually found on the outer surfaces of activated carbons or edges of planes. Silver ions are covalently bonded to the carboxyl group on the porous carbon surface, reduced with NH_4_OH to Ag(0) nanoparticles, and bind with minimal mass transfer resistance. Well-dispersed silver nanoparticles on APC promoted adequate contact with nitroamines as well as strong interaction with APC components, contributing to leaching resistance [38, 39]. After the fifth run of the reduction, the XRD pattern of the used Ag(2)@APC catalyst (Figure 16(b)) was found to be the same as that of the fresh catalyst (Figure 16(a)), confirming that the catalyst structure remained unchanged during the reduction process and Ag(2)@APC catalyst was stable [40]. The reduction efficiency of PNP to PAP is 100% up to the 5^th^ cycle, providing the high stability and reusability of the catalyst.

## 4 Conclusion

We report an effective strategy for the preparation of cost-effective Ag@APC nanoparticles. Ag@APC nanoparticles with face-centered cubic structure, 41-94 nm crystal size, and 1500-1723 m^2^/g BET surface area were obtained by chemical reduction method using NH_4_OH. The catalytic activity of Ag(2)@APC was investigated under different synthesis conditions for PNP, DNP, and TNP reductions and was found to be dependent on initial phenol concentrations and catalyst dosages. The catalytic reduction mechanism was described by the LH model. Ag(2)@APC catalyst showed high catalytic activity towards nitrophenol reductions in short reaction times. The high activity of Ag(2)@APCs was attributed to their large surface area, uniform distribution of Ag nanoparticles within the asphaltene-based porous carbon matrix, and easy transfer of electrons. The catalytic reduction reaction of nitrophenols was accomplished within 4-8 min and is of pseudo-first-order kinetics. The measured rate constants are comparable to or better than the results obtained with the previously synthesized nanocatalysts. The catalyst was easily recovered from the reaction mixture by filtration, and no significant loss of catalyst activity was noted after five runs. No leaching of Ag nanoparticles during the catalytic applications indicates that the catalyst can be reused multiple times and is stable. The synthesized Ag(2)@APC in this study can be suggested as an eco-friendly, low-cost, and efficient catalyst for organic pollutants removal, as well as in other purification processes of industrial applications.

## Declarations

### Author contribution statement

Hikmet Beyza Erdem: Performed the experiments; Analyzed and interpreted the data.

Sevil Çetinkaya: Conceived and designed the experiments; Analyzed and interpreted the data; Contributed reagents, materials, analysis tools or data; Wrote the paper.

### Funding statement

This work was supported by Scientific Research Projects Coordination Unit of 10.13039/100019442Kırıkkale University (Project number: 2019/109).

### Data availability statement

The data that has been used is confidential.

### Declaration of interests statement

The authors declare no conflict of interest.

### Additional information

No additional information is available for this paper.
